# The Diagnostic Pathway Experiences of People Living with Rare Dementia and Their Family Caregivers: A Cross-Sectional Mixed Methods Study Using Qualitative and Economic Analyses

**DOI:** 10.3390/ijerph21020231

**Published:** 2024-02-16

**Authors:** Ian Davies-Abbott, Bethany F. Anthony, Kiara Jackson, Gill Windle, Rhiannon Tudor Edwards

**Affiliations:** 1 The Centre for Applied Dementia Studies, Faculty of Health Studies, University of Bradford, Bradford BD7 1DP, UK; 2 DSDC Wales Research Centre, School of Health Sciences, Bangor University, Bangor LL57 2PZ, UK

**Keywords:** rare dementia, family caregivers, diagnosis, health economics, prevention, pathway, costs, journey

## Abstract

The pathways for receiving a diagnosis of a rare type of dementia are poorly understood. Diagnostic challenges decrease access to relevant health promotion activities and post-diagnostic support. This study was focused on pathways experienced by people affected by rare dementia in Wales, United Kingdom (UK), considering the practical, emotional, and economic consequences. Semi-structured interviews were completed with 10 people affected by rare dementia across Wales, UK (nine family caregivers and one person living with rare dementia). The interview data were subject to a thematic analysis and a bottom-up costing approach was used to cost the pathway journeys. Five transitional points occurred across the diagnostic pathway (initial contact, initial referral, further referrals—provider, further referrals—private, and diagnosis) alongside two further themes (i.e., involved in the diagnostic process and disputes between stakeholders). The timeliness of the diagnosis was perceived to often be subject to ‘luck’, with access to private healthcare a personal finance option to expedite the process. Higher economic costs were observed when, in retrospect, inappropriate referrals were made, or multiple referrals were required. The confusion and disputes relating to individual diagnostic pathways led to further emotional burdens, suggesting that higher economic costs and emotional consequences are interlinked. Clearer diagnostic pathways for rare dementia may prevent unnecessary service contacts, waiting times, and associated distress. Prioritising appropriate and timely service contacts leads to diagnosis and support to families and enables people to increase control over their health. Appropriate diagnostic pathways may be less costly and reduce costs for families.

## 1. Introduction

Worldwide, 55 million people have a diagnosis of dementia [1], and this number is projected to double by 2040 [2]. Only 25% of people living with dementia have received a diagnosis, and 30% are misdiagnosed [1]. In the UK, Wales has a diagnosis rate of 53%, compared to 67% in Northern Ireland, 68% in England, and 73% in Scotland [3]. Alzheimer’s disease is the most prevalent dementia, although vascular and less typical dementia sub-types represent 25% of all diagnoses [4]. People living with rarer forms of dementia are more likely to have a delayed diagnosis, be misdiagnosed [5,6], and are commonly under the age of 65 [7]. The term ‘rare dementia’ describes atypical and inherited types of dementia that are often characterised by a younger onset and cognitive symptoms other than memory loss [4,8] including frontotemporal dementia, primary progressive aphasia, and posterior cortical atrophy. Globally, there are 3.9 million people living with dementia between the ages of 30–60 [9], representing approximately 3% of all people living with dementia. Younger people living with dementia tend to experience additional challenges relating to family, work, and finances [10,11,12,13] and have fewer opportunities for tailored support compared to people with more typical dementia types [14]. An estimated 209, 600 new cases of dementia each year in the UK [2] indicates between 262 to 2358 new cases of rare dementia in Wales alone.

The problem of people living with undiagnosed dementia has been highlighted in UK policies [15,16]. Although UK national guidelines are available regarding the diagnostic process for Alzheimer’s, vascular, Lewy body, and frontotemporal dementia [17], there are no specific pathways for people to receive a diagnosis of other rare types of dementia. Barriers to dementia diagnosis have tended to be explored from the viewpoint of patients and service providers separately. Parker et al.’s (2020) [18] systematic review explored 30 years of patient perspectives, highlighting a lack of knowledge and perceived need alongside denial, fear, and stigma as key barriers to accessing a dementia diagnosis. For service providers, studies suggest that General Practitioners (GPs) perceive time limitations, waiting lists, poor communication between services, inadequate knowledge, and assessment tools as barriers to diagnosis [19,20] Studies concerning the perspective of diagnostic specialist services have tended to emphasise the delivery of a diagnosis [21,22,23] rather than the pathway itself.

The annual healthcare cost of dementia in the UK is £1.7 billion [3]. However, studies exploring the economic cost of dementia have tended to focus on non-medical costs and Alzheimer’s disease [24]. Michalowsky et al.’s (2017) [25] study explored diagnostic costs in a German memory clinic finding that the diagnostic pathway cost increased depending on whether the outcome was mild cognitive impairment (376€/£329.13 converted and inflated to 2021 prices), Alzheimer’s disease (649€/£568.50 converted and inflated to 2021 prices), or vascular/mixed dementia (662€/£579.59 converted and inflated to 2021 prices), although the highest cost was observed in the diagnosis of unspecified dementia types (705€/£617.26 converted and inflated to 2021 prices). In the UK, the overall costs of diagnosis were estimated at £650 per diagnosis (£735.97 when inflated to 2021 prices) [26]. People in Wales with undiagnosed dementia may feel that the emotional and physical cost of pursuing a diagnosis outweighs the benefits of receiving a formal diagnosis [27].

This study explores the experience of people living in Wales who have engaged with diagnostic services to receive a rare dementia diagnosis. It explores the economic cost to both health services and patients, together with the emotional challenges described by participants during the diagnostic process.

## 2. Materials and Methods

### 2.1. Design

A mixed-method cohort study, using qualitative methods and economic analysis was undertaken. Participants were recruited through the Rare Dementia Support (RDS) network as part of a programme approved by the University College London (UCL) research ethics committee (8545/004: Rare Dementia Support Impact Study by Brotherhood et al., 2020) [7].

### 2.2. Participants

Participants were identified through purposive sampling by identifying people in Wales already known to the RDS service [8]. Inclusion criteria for participants were (1) being a person living with rare dementia or a caregiver present during the onset of symptoms and the diagnostic journey, (2) living in Wales, and (3) the capacity to discuss their experience in a semi-structured interview.

An email describing this study and inviting participation was sent to 55 members living in Wales, with two follow-up invitations if there was no response. Ten people were interviewed (one person living with rare dementia and nine family members) between August 2020 and May 2022 with interviews lasting between 33 and 97 min.

### 2.3. Procedure

Participants were provided with an information sheet and the opportunity to discuss any concerns prior to providing consent. Interviews were conducted using a semi-structured design. Fifty-two questions were devised using Kallio et al.’s (2016) [28] semi-structured interview guide by using the information gathered through a brief scoping review regarding rare dementia diagnostic pathways in Wales (interview questions are presented in Appendix A). Telephone interviews were used due to participants being located across Wales and social distancing measures inhibiting the opportunity for face-to-face interviews.

### 2.4. Analysis

Thematic analysis was used to explore the narratives in-depth and to allow them to be honestly presented without analytical manipulation using Braun and Clarke’s framework [29,30]. Interviews were listened to several times and transcribed verbatim. Initial coding was developed using an inductive approach. As the codes were analysed and the diagnostic process became clearer, themes evolved into linear transitional points within the diagnostic journey. The remaining codes and themes were further defined, redefined, and evolved into supplementary themes alongside the transitional points. Whilst each narrative was situationally distinct, the thematic similarities between participant pathways indicate that data saturation was achieved.

A bottom-up costing approach was used to cost the pathway journeys [31]. Costs were presented in British Pounds Sterling (£) for the cost year 2021. The unit cost schedule costs included in the analysis (including sources of costs and costing assumptions) can be viewed in Appendix B. Costs are broken down for each stage of the pathway journey with total cost rows rounded to the nearest whole pound (£). The economic analysis was conducted from a National Health Service (NHS) and wider patient perspective, including costs directly incurred by the patient through private consultations and opportunity costs of potential lost income from attending appointments and investigations [32]. The NHS is a publicly funded healthcare system. Patients entitled to NHS treatment may choose to pay to access NHS or private healthcare providers, usually to avoid waiting lists or to access treatment unavailable from the NHS [33]. Consultation time information used to calculate opportunity costs for patients attending consultations was based on the consultation duration information provided in the published unit costs (Appendix B), apart from one instance, where a participant provided information on the duration of their appointment. Opportunity costs for travel time attending appointments were based on the patient receiving the services i.e., the travel time for one person. Lost income was valued at the UK national living wage in 2021 (£8.91 per hour for people aged 23 and over in April 2021 [34]). Travel time and mileage costs were also calculated if travel duration and mileage information were provided by participants. A sensitivity analysis was conducted to show the difference in opportunity costs from lost leisure time valued at £14.82 per hour in the cost year 2021, converted and inflated from 16 Euros per hour in 2014 from published estimates [35]. Sensitivity analysis result tables are available in Appendix C.

## 3. Results

Nine participants were family caregivers, and one was a person living with a rare dementia. Seven of the participants were female and three were male, including the person living with dementia. The ages of the participants ranged from 42 to 85, with a mean of 62 and a median of 72. The person living with dementia had a diagnosis of semantic dementia, a variant of frontotemporal dementia. Their capacity to consent was assessed as dictated by the Mental Capacity Act 2005 [36] by the lead researcher, a registered nurse in mental health. Participants shared experiences relating to the diagnosis of frontotemporal dementia (6), primary progressive aphasia (3), and posterior cortical atrophy (1). All participants were White British.

The thematic analysis revealed two distinct experiential themes concerning how the person felt involved in the diagnostic process and their experience of disputes between stakeholders. These occurred alongside five transitional points across the diagnostic pathways of people living with rare dementia:

1. Initial contact
2. Initial referral
3. Further referrals—provider
4. Further referrals—private
5. Diagnosis

### 3.1. Involved in the Diagnostic Process

Caregivers often felt excluded from the diagnostic process and the sharing of the diagnosis. One caregiver was excluded from the diagnosis meeting despite her husband’s limited retention and understanding of the meeting:

‘I can’t understand when you go into a consultant, and you’ve got someone who’s got memory problems and speech problems, and yet the partner, the wife, isn’t allowed’. (Participant 1)

This exclusion was felt when the diagnosis was impersonally delivered, through a letter rather than a conversation. Other participants were less concerned about their involvement in the process, regarding themselves as an object to be examined rather than an active participant:

‘I turned up, I submitted myself to whatever they wanted to do. From an engineering point of view, I wasn’t part of the plan’. (Participant 2)

The sense of being uninvolved was also related to caregivers having to ‘fight’ and ‘chase’ clinicians for appointments and referrals. This was not regarded as positive involvement in the pathway to diagnosis but a necessary action to ensure that the diagnosis continued to move forward:

‘I’m always double-checking things now, if a referral has been made. And I’m ringing up and chasing’. (Participant 1)

Participants also raised larger concerns about what happened to people who lacked the ability to ‘chase’ and fight’:

‘Those who do not push hard enough will just drop into an abyss’. (Participant 4)

Whilst all participants felt that their inclusion in the diagnostic process was not automatically assumed by services, there were differing opinions on whether their involvement was integral or inconsequential to the progression of the pathway.

### 3.2. Disputes between Stakeholders

Several caregivers described disputes between themselves and the person living with rare dementia. These were often caused by a lack of insight into behavioural and cognitive changes, resulting in a refusal to seek help:

‘My wife wouldn’t co-operate. She wasn’t being bolshy or difficult, but she could not see why we wanted her to see a doctor’. (Participant 7)

Some participants felt that GPs were unsure about who they should refer to, with services disputing that the referral was appropriate:

‘But then, he rang back and said that he’d contacted the memory clinic and the memory clinic, had said that he is too young, to be referred to somewhere like that’. (Participant 1)

Disputes led to extra costs, as participant 1′s difficulties in securing services led to a personal outlay of £895 for private consultations. Caregivers also felt that they were expected to align with the service provider’s expectations rather than the service adapting to meet the needs of the person. One person’s assessment was delayed due to the service only offering clinic-based appointments. The caregiver described their husband’s refusal to leave the house, as part of his symptoms, but still be expected to attend the clinic:

‘They wouldn’t come here to us. We have, and I have pleaded with them, look I can’t get him out and they’ve, no, he’s got to go, you’ve got to get him there’. (Participant 6)

The emotional impact of dealing with an unhelpful service alongside her husband’s symptoms also resulted in participant 6 relying on private healthcare to expedite the diagnostic process. The caregiver found that after being informed that a psychiatry appointment by a referring psychologist was urgent, they had been placed at the bottom of the waiting list:

‘The psychologist had said I’m going to pass him over as an urgent patient. And I heard nothing and when I rang to speak to the secretary to find out where he was on the list, they said, oh, the doctor said it’s not an emergency’. (Participant 6)

Whilst some participants felt that a formal diagnosis of dementia provided some relief, as they now understood what was happening, others continued to experience disputes between clinicians regarding the accuracy of the diagnosis. These disputes prevented some participants from accessing care services and elongated the emotional cost of the diagnostic pathway.

1. Initial contact

Table 1 and Table 2 provide a breakdown of the initial contact costs and initial referral costs, respectively. Initial contact costs for nine participants were funded by the NHS (healthcare system in the United Kingdom), and one participant reported an initial referral that was privately funded by the patient/family (Table 1). Initial contact was typically with a GP, with exceptions noted in Table 1. The least costly consultation was a GP appointment (£39.23), whereas the highest costing consultation was a privately funded consultant neurologist appointment valued at £250 (Table 1). For the ten participants, the total cost for the initial contact was £811 (£561 for NHS costs) when excluding travel and opportunity costs. When opportunity costs for patient consultation time, travel time, and mileage costs were considered, the total cost for initial contact was £911 (Table 1). Only one participant (participant 3) reported the length of time that they attended the initial contact appointment (480 min, Table 1) and this was acknowledged as an outlier in the data. For the other initial contacts reported by participants, a 9.22-min consultation time was assumed for GP appointments, and a 60-min consultation time for all other contacts was based on the consultation duration information provided in the published unit costs (Appendix B). When excluding this outlier of 480 min reported by participant 3, the total cost for initial contact (including opportunity costs) would be £839.72. There was more diversity in initial referrals, with some people referred for scans whilst others went directly to assessing clinicians (Table 2). All the initial referral contacts for the ten participants were funded by the NHS (Table 2). Consultation with the community mental health nurse was the least costly initial referral (£44), and a memory clinic appointment had the highest cost (£528).

2. Initial referral

**Table 2:** Initial referral costs.

Participant	Initial Referral (NHS/Private)	Health Service Activity	Unit Cost	Consultation Time (Minutes)	Travel Time (Minutes)	Opportunity Costs—Patient Consultation Time (£)	Opportunity Costs—Patient Travel Time (£)	Patient Funded Costs—Mileage (£0.45 per Mile)	Total Costs (£)
Participant 1	NHS	CT scan	£70.00	60	20	£8.91	£2.97	**	£82
Participant 2	NHS	MRI scan	£110.00	60	**	£8.91	**	**	£119
Participant 3	NHS	Ophthalmology follow-up appointment	£139.23	240 ***	30	£35.64	£4.46	**	£179
Participant 4	NHS	Community mental health nurse contact	£44.00	60	**	£8.91	**	**	£53
Participant 5	NHS	CT scan	£70.00	60	**	£8.91	**	**	£79
Participant 6	NHS	Community mental health nurse contact	£44.00	60	**	£8.91	**	£1.35	£54
Participant 7	NHS	Well woman appointment	£44.00	60	**	£8.91	**	**	£53
Participant 8	NHS	Memory clinic appointment	£528.00	60	**	£8.91	**	£6.30	£543
Participant 9	NHS	Psychiatrist consultation	£120.00	60	**	£8.91	**	£1.80	£131
Participant 10	NHS	CT scan	£70.00	60	**	£8.91	**	**	£79
**Totals:**			**£1239**	**780**	**50**	**£116**	**£7**	**£9**	**£1372**

(**) Missing data. (***) Consultation time information provided by the participant.

3. Further referrals—provider

Table 3 provides the cost breakdown of further NHS referrals and activity following the initial contact and referral. The total costs to the NHS for referrals and activity following the initial contact for the ten participants were £10,854 (Table 3).

4. Further referrals—private

The decision to access private care was typically based on the potential to expedite the diagnostic process but also dependent on the person’s financial or insurance position:

‘Fortunately, he had a work cover on an insurance policy, which meant he could get a faster appointment with the neurologist…if we had to wait 18 months or more, you know, we still wouldn’t have known what was going on’. (Participant 5)

Table 4 provides the costs of private consultations (directly incurred by the patient) following the initial contact and referral. Following the initial contact, five participants resorted to paying privately for further investigations and consultations with clinicians at a total consultation cost of £2440 (Table 4). When also considering travel and opportunities, the total cost was £2562 (Table 4).

5. Diagnosis

Many caregivers described being shocked by the diagnosis, but upon reflection, they were angry about the lack of or quality of information provided:

‘I wish to stress that very clearly, I am very annoyed that I wasn’t given more information’. (Participant 8)

Receiving a diagnosis was perceived by several participants as ‘luck’, encountering a clinician with knowledge about rare dementia rather than due to a clear pathway. Table 5 provides the total costs of the diagnostic pathway. Participant 10 had the lowest costing pathway journey at a total cost of £503, whereas participant 2 had the highest costing pathway journey at a total cost of £8043 (Table 5). The average total pathway cost across the ten participants was £1636 (Standard Deviation (SD) 2288.53). When excluding the outlier of 480 min (reported by participant 3) in our opportunity cost calculation for patient consultation time, the average total pathway cost across the ten participants was £1629 (2290.37).

Table 6 provides a breakdown of resource use costs along the pathways to diagnosis from the healthcare system (NHS) and patient (privately funded by families) perspectives. In terms of total NHS resource use across the pathways to diagnosis, the ten participants reported a total of 73 separate resource use items, at an average cost per item of £173.33 (SD 180.14). Of the participants who had reported resource use that was privately funded by the patient/family, 12 separate resource use items were reported at an average cost per item of £224 (SD193.89). The NHS and privately funded resource use items with the highest cost were memory clinic appointments (£528) and CT scans (£695), respectively (Table 6).

## 4. Discussion

The shared narratives illustrated an emotional cost through the variation in the diagnostic process, with some participants emotionally suffering more than others, as evidenced in the two themes. The barriers to diagnosis shared by participants were similar to previous studies about the diagnosis of dementia [18,19,20], including poor communication between stakeholders, disputes regarding referrals to services, or the diagnosis itself. Inadequate GP knowledge led to recurring visits to initiate a referral or inappropriate initial referrals. This corresponds to the experience of people living with young onset dementia in Canada whose diagnosis was delayed as healthcare professionals attributed their symptoms to causes other than dementia, which resulted in extended pathways of tests and travel [37].

The emotional and economic costs of the pathway are interlinked, as costs were seen to rise when the pathway to diagnosis was unclear, resulting in more referrals and disputes. Unlike people navigating the established diagnostic pathways for dementia, participants perceived the eventual diagnosis as the result of ‘luck’ rather than a clear pathway. As indicated in Table 5, the highest total diagnostic pathway cost was £8043 (participant 2) and the lowest total diagnostic pathway cost was £462 (participant 4). The variation in the economic cost between participants is indicative of this perceived ‘luck’ as three people received a timely diagnosis at an economic cost of £462, £503, and £570, which is lower than the diagnosis cost of £617.26 (inflated to 2021 prices) reported in Michalowsky et al.’s (2017) [25] study for people with unspecified dementia. However, of the people receiving this more timely and less challenging diagnosis, only participant 7 was able to do so without accessing private healthcare, suggesting that a personal economic burden is required for a timely diagnosis of rare dementia. Although participant 4 was perceived to have a comparatively straightforward diagnosis with a low economic cost, potentially supporting the role of a community nurse as an initial referral, it should be noted that more than 50% (£250 of £462) of the overall cost was privately funded by the patient/family. A total cost of £2690 for private consultations was paid between six participants. ‘Luck’ in dementia services aligns with research indicating that a ‘postcode lottery’ exists concerning post-diagnostic information and support depending on location [38,39]. The overall experience of receiving post-diagnostic information in this study was regarded as poor.

The economic and emotional impact of the pathways was illustrated when participant 6 was expected to bring their husband to a clinical appointment despite his contrary symptoms. The economic cost to the NHS increased as appointments were arranged, cancelled, or unattended. The economic cost to the participant was observed as opportunity costs increased due to the time spent in dispute, which led to further anxiety alongside his existing symptoms. The lack of knowledge in the service corresponds to other studies [18,19,20] observing a delayed diagnosis and access to support services.

A delay in post-diagnostic support was also perceived in participant 1’s experience when they were excluded from the diagnostic meeting. The perception of feeling uninvolved results in inadequate post-diagnostic support. Positive post-diagnostic support includes specialist advice and opportunities to have a voice and a consideration of family relationships [40]. The perceived ‘luck’ of rare dementia diagnosis suggests that positive examples of support are uncommon in Wales. However, whilst ‘luck’ may appear to be an unexpected quality of receiving a diagnosis of any type of dementia, the perspectives of people in this study suggest that, unlike a previous study in Wales [27], pursuing and receiving a formal diagnosis outweighs the emotional and physical cost of the pathway.

### Limitations of This Study

Whilst 10 participants limit the transferability of the economic findings, this study included a hard-to-reach population in Wales and is a novel achievement. The smaller number also supported an in-depth qualitative analysis. Gathering data using semi-structured interviews allowed the narratives of participants to be heard [41,42], providing reliable and comparable qualitative results [42]. Methodologically, telephone interviews were sound as these aid agenda-driven questioning [43] and are a viable method to collect rich qualitative data on sensitive subjects [29].

This study only included people and family caregivers who had a confirmed diagnosis of rare dementia. Including people who had received similar assessments but had not received a rare dementia diagnosis may have illustrated different perspectives of diagnostic pathways.

## 5. Conclusions

There are substantial costs to a rare dementia diagnosis, although many of these are wasted as economic costs and emotional burdens rise due to disputes and confusion over the diagnostic process. The education of primary care referrers regarding rare dementia may reduce some of the inappropriate referrals perceived in this study. However, this study has illustrated that referrals to psychiatric services may not immediately lead to a diagnosis, depending on the knowledge of the clinician. Pathways for more typical forms of dementia indicate the services expected to provide a diagnosis and crucially, diagnostic support. Whilst clearer pathways are required, establishing which clinical area should provide the diagnosis and post-diagnostic support should encourage the development of education programmes in those settings by providing clinicians with better knowledge about rare dementia and alleviate concerns regarding whether the pathway is worth taking for people living with rare dementia and their families. Little is known about pathways to rare dementia diagnoses in Wales, and the findings of this pilot work can support policy documents and the design of services to acknowledge the process that patients go through to achieve a diagnosis.

## Figures and Tables

**Table 1:** Initial contact costs.

Participant	Initial Contact (NHS/Private)	Health Service Activity	Unit Cost	Consultation Time (Minutes)	Travel Time (Minutes)	Opportunity Costs—Patient Consultation Time (£)	Opportunity Costs—Patient Travel Time (£)	Patient Funded Costs—Mileage (£0.45 per Mile)	Total Costs (£)
Participant 1	NHS	GP consultation	£39.23	9.22	**	£1.37	**	**	£41
ParticipantT 2	NHS	Neurologist consultation	£120.00	60	**	£8.91	**	**	£129
Participant 3	NHS	Ophthalmology appointment	£165.95	480 ***	30	£71.28	£4.46	**	£242
Participant 4	Private	Consultant neurologist appointment	£250.00	30	**	£4.46	**	**	£254
Participant 5	NHS	GP consultation	£39.23	9.22	**	£1.37	**	**	£41
Participant 6	NHS	GP consultation	£39.23	9.22	**	£1.37	**	£1.35	£42
Participant 7	NHS	GP consultation	£39.23	9.22	**	£1.37	**	**	£41
Participant 8	NHS	GP consultation	£39.23	9.22	**	£1.37	**	**	£41
Participant 9	NHS	GP consultation	£39.23	9.22	**	£1.37	**	**	£41
Participant 10	NHS	GP consultation	£39.23	9.22	**	£1.37	**	**	£41
Total cost for NHS consultations:			£561						
Total cost for private consultations:			£250						
**Totals:**			**£811**	**635**	**30**	**£94**	**£4**	**£1**	**£911**

(**) Missing data. (***) Consultation time provided by the participant.

**Table 3:** Further NHS referral and activity costs.

Participant	Health Service Activity	Unit Cost	Consultation Time (Minutes)	Travel Time (Minutes)	Opportunity Costs—Patient Consultation Time (£)	Opportunity Costs—Patient Travel Time (£)	Patient Funded Costs—Mileage (£0.45 per Mile)	Total Costs (£)
Participant 1	GP telephone call	£15.52	**	na	**	na	na	£16
	Memory service telephone call	£8.67	**	na	**	na	na	£9
	Consultant neurologist consultation	£123.00	60	**	£8.91	**	**	£132
Participant 2	Psychologist consultation	£65.00	60	**	£8.91	**	**	£74
	Memory service appointment (June 2015)	£528.00	60	40	£8.91	£5.94	**	£543
	Memory service appointments ×12 (Dec 2015–June 2021)	£6336.00	720	480	£106.92	£71.28	**	£6514
	SPECT scan	£243.16	60	120	£8.91	£17.82	**	£270
	MRI scan	£110.00	60	120	£8.91	£17.82	**	£137
	Consultation with consultant Psychologist and consultant Psychiatrist	£243.00	60	40	£8.91	£5.94	**	£258
ParticipantT 3	MRI scan	£110.00	60	**	£8.91	**	**	£119
	Ophthalmology appointment (follow-up)	£139.23	60	30	£8.91	£4.46	**	£153
	Ophthalmology appointment (follow-up)	£139.23	60	30	£8.91	£4.46	**	£153
	GP consultation	£39.23	9.22	**	£1.37	**	**	£41
	Neurologist consultation	£120.00	60	30	£8.91	£4.46	**	£133
Participant 4	Levadopa Parkinson’s medication (2-week trial)	£7.98	na	na	na	na	**	£8
	Neurologist consultation	£120.00	60	120	£8.91	£17.82	**	£147
Participant 5	-	-	-	-	-	-	-	-
Participant 6	GP consultation	£39.23	9.22	**	£1.37	**	£1.35	£42
	Psychologist consultation	£65.00	60	**	£8.91	**	£6.30	£80
	MRI scan	£110.00	60	**	£8.91	**	£7.20	£126
	Psychiatrist consultation	£120.00	60	**	£8.91	**	£7.20	£136
	Psychiatrist phone call	£8.67	**	na	**	na	na	£9
Participant 7	Psychiatrist consultation	£120.00	60	**	£8.91	**	**	£129
	MRI scan	£110.00	60	**	£8.91	**	£39.60	£159
	Psychiatrist consultation	£120.00	60	10	£8.91	£1.49	**	£130
	Mental health nurse	£41.00	60	**	£8.91	**	**	£50
	Donepezil 5 mg (1 day)	£0.04	na	na	na	na	na	£0
	Psychiatrist phone call	£8.67	**	na	**	na	na	£9
Participant 8	CT scan	£70.00	60	**	£8.91	**	**	£79
	Consultant Psychiatrist consultation	£123.00	60	**	£8.91	**	£8.10	£140
Participant 9	Psychologist consultation	£65.00	60	**	£8.91	**	**	£74
	Psychologist consultation	£65.00	60	**	£8.91	**	**	£74
	Psychiatrist consultation (×8)	£960.00	480	**	£71.28	**	£14.40	£1046
	Psychologist home visit (2 h)	£130.00	120	**	£17.82	**	**	£148
	MRI scan	£110.00	60	**	£8.91	**	**	£119
	Psychiatrist consultation	£120.00	60	**	£8.91	**	£1.80	£131
Participant 10	Neurologist consultation	£120.00	60	**	£8.91	**	**	£129
**Totals:**		**£10,854**	**2838**	**1020**	**£422**	**£151**	**£86**	**£11,513**

(**) Missing data. (na) Not applicable. (-) No consultations were reported.

**Table 4:** Further private consultation and activity costs.

Participant	Health Service Activity	Unit Cost	Consultation Time (Minutes)	Travel Time (Minutes)	Opportunity Costs—Patient Consultation Time (£)	Opportunity Costs—Patient Travel Time (£)	Patient Funded Costs—Mileage (£0.45 per Mile)	Total Costs (£)
Participant 1	Neurologist consultation	£250	30	120	£4.46	£17.82	**	£272
	MRI scan	£395	60	120	£8.91	£17.82	**	£422
	Neurologist online consultation (Zoom)	£250	30	na	£4.46	na	na	£254
Participant 2	-	-	-	-	-	-	-	-
Participant 3	Optician eye test ×2	£50	60	**	£8.91	**	**	£59
	Optician eye test ×2	£50	60	**	£8.91	**	**	£59
Participant 4	-	-	-	-	-	-	-	-
Participant 5	Neurologist consultation	£250	30	**	£4.46	**	£10.80	£265
	Neurologist consultation	£250	30	**	£4.46	**	£10.80	£265
Participant 6	CT scan	£695	60	**	£8.91	**	£7.20	£711
Participant 7	-	-	-	-	-	-	-	-
Participant 8	-	-	-	-	-	-	-	-
Participant 9	-	-	-	-	-	-	-	-
Participant 10	Neurologist consultation	£250	30	**	£4.46	**	**	£254
**Totals:**		**£2440**	**390**	**240**	**£58**	**£36**	**£29**	**£2562**

(**) Missing data. (na) Not applicable. (-) No consultations were reported.

**Table 5:** Total costs of diagnosis pathway.

Patient	NHS Costs (£)	Patient Funded Costs—Private Consultations (£)	Travel Time (Minutes)	Consultation Time (Minutes)	Opportunity Costs—Travel Time (£)	Opportunity Costs—Patient Consultation Time (£)	Patient Funded Costs—Mileage (£)	Total Costs (£)
Participant 1	£256.42	£895.00	260	249.22	£38.61	£37.02	**	£1227
Participant 2	£7755.16	£0.00	800	1140	£118.80	£169.29	**	£8043
Participant 3	£852.87	£100.00	150	1089.22	£22.30	£161.75	**	£1137
Participant 4	£171.98	£250.00	120	150	£17.82	£22.28	**	£462
Participant 5	£109.23	£500.00	**	129	**	£19.20	£21.60	£650
Participant 6	£426.13	£695.00	**	318	**	£47.29	£31.95	£1200
Participant 7	£482.94	£0.00	10	309.22	£1.49	£45.92	£39.60	£570
Participant 8	£760.23	£0.00	**	189.22	**	£28.10	£14.40	£803
Participant 9	£1609.23	£0.00	**	909.22	**	£135.02	£18.00	£1762
Participant 10	£229.23	£250.00	**	159.22	**	£23.65	**	£503
**Total**	**£12,653**	**£2690**	**1340**	**4643**	**£199**	**£690**	**£126**	**£16,357**

** Missing data.

**Table 6:** Resource use costs along the pathways to diagnosis from the healthcare system (NHS) and patient (privately funded) perspectives.

Resource Use	Number of Participants Reporting Resource Use	Total Cost	Total Number of Resource Use Items Reported by Participants	Lowest Cost Resource Use Item Reported	Highest Cost Resource Use Item Reported	Mean Cost of Service Use Items Reported	Standard Deviation (SD)
Initial contact—NHS	9	£561	9	GP consultation (£39.23)	Ophthalmology appointment (£165.95)	£62.28	47.17
Initial contact—Private	1	£250	1	na	na	na	na
Initial referral—NHS	10	£1239	10	Community mental health nurse contact (£44)	Memory clinic appointment (£528)	£123.92	145.91
Initial referral—Private	0	na	na	na	na	na	na
Further referrals/activity—NHS	9	£10,854	54	Donepezil 5 mg (£0.04)	Memory clinic appointment (£528)	£201	191.83
Further referrals/activity—Private	5	£2440	11	Optician eye test (£25)	CT scan (£695)	£222	203.18
Total NHS resource use across pathways	10	£12,653	73	Donepezil 5 mg (£0.04)	Memory clinic appointment (£528)	£173.33	180.14
Total privately funded resource use across pathways	6	£2690	12	Optician eye test (£25)	CT scan (£695)	£224	193.89

(na) Not applicable.

## Data Availability

The datasets used and/or analysed during the current study are available from the corresponding author upon reasonable request.

## Appendix A

Interview Questions

Q1. At the onset of symptoms, what did you feel was happening? Did you have any alternative explanations for the symptoms?
Q2. Did you discuss your symptoms with anybody else prior to contacting services? e.g., family, friends, and colleagues
Q3. Did the symptoms affect any of your relationships? Family relationships? Working relationships?
Q4. Did the symptoms have any financial, or other, implications?
Q5. Did you have any concerns about approaching health services to discuss your symptoms? e.g., concerns about stigma
Q6. Who did you first approach regarding your concerns? (e.g., a health provider, social services, third sector organisation) Year? Language? If a health provider is the first contact, go to Q.8
Q7. How soon after this initial contact did you first have contact with health services regarding your concerns? Year? Language Length of appt?
Q8. Did you feel confident the health service would be able to help you?
Q9. During or immediately following this first contact with health services, were you referred to a diagnostic service? Year? If yes, go to Q.10 If no, go to Q.14
Q10. Which service were you referred to? e.g., Memory service, neurology, or psychology
Q11. How long did you remain on a waiting list before being seen by the service?
Q12. How far was the service geographically located from your home address? Request location of service if distance is unknown
Q13. What mode of transport did you use to attend this service? Go to Q. 23
Q14. Did you need to make further contact with health services to initiate a referral to a diagnostic service? Year?
Q15. How long did you have to wait between your initial contact with health services and a referral being made?
Q.16 Who completed the referral to the diagnostic service when it happened?
Q.17 Which service were you referred to? e.g., Memory service, neurology, or psychology
Q.18 How long did you remain on a waiting list before being seen by the service?
Q.19 How far was the service geographically located from your home address? Request location of service if distance is unknown
Q20. What mode of transport did you use to attend this service?
Q.21 Were you referred to any support services between the time of first contact and receiving your diagnosis? If YES, request the details of these services. Year? Sessions attended?
Q.22 Have you at any point received an assessment of your needs as a carer?
Q.23 How long did you wait for your diagnosis following your first contact with the diagnostic service?
Q.24 How involved were you in the diagnostic process?
Q.25 Who was the first to think this might be a rare dementia?
Q. 26 If not by the professional, please specify how you/others came to think it was a rare dementia.
Q.27 Who told you about the diagnosis? Year? Length of appt?
Q.28 How much do you agree or disagree that you knew what was going to happen after the diagnosis?
Q. 29 Do you think the diagnosis of dementia, for the person you care for, was delayed because of other pre-existing conditions?
Q.30 Which conditions do you think delayed diagnosis?
Q.31 Has the person you care for had any additional conditions diagnosed since their dementia diagnosis?
Q.32 If applicable: Thinking about the additional condition(s), do you feel that the diagnosis was delayed, or complicated, due to the existing dementia diagnosis?
Q. 33 Do you feel that the overall treatment of any additional conditions for the person with dementia has changed since their dementia diagnosis?
Q.34 How did you feel about the way you were given your diagnosis?
Q.35 How did you feel about the diagnosis?
Q.36 What information were you given about the diagnosis?
Q.37 As a carer, how would you rate the quality of information you have received from professionals since the diagnosis?
Q38 Did you feel that you understood the diagnosis after the health provider explained what it was?
Q.39 Were you and the person you care for able to have a practical conversation about the future following their dementia diagnosis?
Q.40 Immediately following diagnosis, what interventions were discussed and/or initiated? Use the list below to support the participant in recalling these possible interventions. Medication Activities for the person living with rare dementia Carer support Befriending services Carer education Day services Cognitive stimulation therapy Cognitive rehabilitation Other
Q41. When did you become aware of Rare Dementia Support?
Q42. How did you become aware of Rare Dementia Support?
Q.43 Since diagnosis, have you been referred to any other services? Use the list below to support the participant to recall these possible interventions. Medication Activities for the person living with rare dementia Carer support Befriending services Carer education Day services Cognitive stimulation therapy Cognitive rehabilitation Other
Q.44 What support, if any, do you feel would have made the greatest difference to both you as a carer and the PLWD?
Q.45 How much do you agree that the following are important in helping to manage the symptoms of dementia? - good nutrition - being well hydrated - physical therapy
Q.46 Do you remain in contact with either the diagnostic service or another health provider regarding your rare dementia diagnosis? If yes, go to Q.47 If no, go to Q. 48
Q.47 How often do you have contact with this service and by what means? Use the list below to support the participant. Go to Q.52 Face-to-face appointments Telephone appointments Email appointments Other
Q.48 If you require support from health services, do you know how to access this?
Q.49 From your previous experiences, do you feel that support would be provided in a timely manner?
Q.50 Overall, how would you describe your journey from the initial recognition of symptoms to receiving a diagnosis of a rare dementia?
Q.51 Taking everything into account, how satisfied or dissatisfied are you with the support you have received since the diagnosis?
Q.52 What changes would you suggest to services for people affected by rare dementia in Wales?

## Appendix B

Unit Cost Schedule for the Economic Analysis.

**Primary Care and Community-Based Consultations**	**Unit Cost**	**Source**	**Notes/Assumptions**	**Citation/Website Link**
GP consultation	£39.23	PSSRU Unit costs of health and social care 2021 [44]	Including direct care staff costs and qualification costs	Jones KC, Burns A. Unit costs of health and social care 2021. p. 111
GP telephone call	£15.52	PSSRU Unit costs of health and social care [44]	Cost per intervention. GP-led telephone triage. Cost per intervention, including other costs	Jones KC, Burns A. Unit costs of health and social care 2021. p.114
Community mental health nurse appointment	£44	PSSRU Unit costs of health and social care [44]	Cost per working hour. Band 5	Jones KC, Burns A. Unit costs of health and social care 2021. p.108
Well woman appointment	£44	PSSRU Unit costs of health and social care [44]	Cost per working hour. Practice nurse.	Jones KC, Burns A. Unit costs of health and social care 2021. p.109
Community mental health nurse appointment	£44	PSSRU Unit costs of health and social care [44]	Cost per working hour. Band 5	Jones KC, Burns A. Unit costs of health and social care 2021. p.108
**Hospital Attendances and Related Activity**	**Unit Cost**	**Source**	**Notes/Assumptions**	
Neurologist consultation	£120	PSSRU Unit costs of health and social care [44]	Cost per working hour. Associate specialist.	Jones KC, Burns A. Unit costs of health and social care 2021. pp.139–141
Consultant neurologist consultation	£123	PSSRU Unit costs of health and social care [44]	Cost per working hour. Consultant: medical	Jones KC, Burns A. Unit costs of health and social care 2021. pp.139–141
Ophthalmology appointment (first attendance)	£165.95	National Schedule of NHS costs 2020-21 [45]	Currency code WF01B. Non-consultant led. Non-Admitted Face-to-Face Attendance, First.	https://www.england.nhs.uk/publication/2020-21-national-cost-collection-data-publication/ (accessed on 1 November 2023)
Ophthalmology appointment (follow-up)	£139.23	National Schedule of NHS costs 2020-21 [45]	Currency code WF01A. Non-consultant led. Non-Admitted Face-to-Face Attendance, Follow-up.	https://www.england.nhs.uk/publication/2020-21-national-cost-collection-data-publication/ (accessed on 1 November 2023)
CT scan	£70	NHS National tariff workbook 2020/21 (Annex A). [46]	HRG code: RD20A. Computerised Tomography Scan of One Area, without contrast, 19 years and over. NHS and private investigations (CT, MRI, and SPECT scans) were assumed to last 60 min [47]	https://www.england.nhs.uk/wp-content/uploads/2021/02/20-21NT_Annex_A_National_tariff_workbook.xlsx (accessed on 1 November 2023) https://www.cuh.nhs.uk/patient-information/information-for-patients-who-are-having-a-ct-scan/ (accessed on 1 November 2023)
MRI scan	£110	NHS National Tariff Workbook 2020/21 (Annex A). [46]	HRG code: RD01A. Magnetic Resonance Imaging Scan of One Area, without contrast, 19 years and over. NHS and private investigations (CT, MRI, and SPECT scans) were assumed to last 60 min [47]	https://www.england.nhs.uk/wp-content/uploads/2021/02/20-21NT_Annex_A_National_tariff_workbook.xlsx (accessed on 1 November 2023) https://www.cuh.nhs.uk/patient-information/information-for-patients-who-are-having-a-ct-scan/ (accessed on 1 November 2023)
SPECT scan	£243.16	National Schedule of NHS costs 2020/21 [45]	Currency code: RN08A. Single Photon Emission Computed Tomography (SPECT), 19 years and over. NHS and private investigations (CT, MRI, and SPECT scans) were assumed to last 60 min [47]	https://www.england.nhs.uk/publication/2020-21-national-cost-collection-data-publication/ (accessed on 1 November 2023) https://www.cuh.nhs.uk/patient-information/information-for-patients-who-are-having-a-ct-scan/ (accessed on 1 November 2023)
Memory service appointment	£528	PSSRU Unit costs of health and social care [44]	Total cost per hour.	Jones KC, Burns A. Unit costs of health and social care 2021. p.28
Memory service telephone call	£8.67	See notes. PSSRU Unit costs of health and social care [44]	No cost data available—costed as GP telephone call in response to e-consultation.	Jones KC, Burns A. Unit costs of health and social care 2021. p.28
Psychologist consultation	£65.00	PSSRU Unit costs of health and social care [44]	Cost per working hour. Band 7 clinical psychologist.	Jones KC, Burns A. Unit costs of health and social care 2021. pp.102–104
Psychiatrist consultation	£120	PSSRU Unit costs of health and social care [44]	Per working hour. Associate specialist.	Jones KC, Burns A. Unit costs of health and social care 2021. pp.139–141
Consultant psychologist consultation	£120	PSSRU Unit costs of health and social care [44]	Clinical psychologist consultant (Band 8d)	Jones KC, Burns A. Unit costs of health and social care 2021. pp.133–135
Consultation psychiatrist consultation	£123	PSSRU Unit costs of health and social care [44]	Cost per working hour for consultant psychiatric	Jones KC, Burns A. Unit costs of health and social care 2021. p.141
Psychiatrist phone call	£8.67	See notes. PSSRU Unit costs of health and social care [44]	No cost data available—costed as GP telephone call in response to e-consultation.	Jones KC, Burns A. Unit costs of health and social care 2021. p.28
Psychologist home visit	£65.00	See notes. PSSRU Unit costs of health and social care [44]	No unit cost data available. Costed as psychologist cost per working hour	Jones KC, Burns A. Unit costs of health and social care 2021. pp.102–104

**Private Consultations**	**Unit Cost**	**Source**	**Notes/Assumptions**	**Link**
Neurologist and consultant neurologist consultation	£250	Nuffield Health Hospital guide price [48]	30-min appointment. Nuffield Health—Nuffield Health Grosvenor (Chester) Hospital consultation price	https://www.nuffieldhealth.com/consultants?size=n_20_n&sort-field=sortname&sort-direction=asc (accessed on 1 November 2023)
Neurologist online consultation (Zoom)	£250	See notes section. Nuffield Health hospital guide price [48]	No data cost data available. Costed as neurologist face-to-face consultation	https://www.nuffieldhealth.com/consultants?size=n_20_n&sort-field=sortname&sort-direction=asc (accessed on 1 November 2023)
MRI scan	£395	Nuffield Health—Cardiff and Vale hospital guide price [48]	MRI scan (1 part). Price includes pre-assessment, main treatment, and post-discharge care. Initial consultation from £0. All scans assumed to last 60 min [47]	https://www.nuffieldhealth.com/hospitals/cardiff-and-vale/pricing (accessed on 1 November 2023) https://www.cuh.nhs.uk/patient-information/information-for-patients-who-are-having-a-ct-scan/ (accessed on 1 November 2023).
CT scan	£695	Nuffield Health—Cardiff and Vale hospital guide price [48]	CT scan (1 part). Price includes pre-assessment, main treatment, and post-discharge care. Initial consultation from £0. All scans were assumed to last 60 min [47]	https://www.nuffieldhealth.com/hospitals/cardiff-and-vale/pricing (accessed on 1 November 2023) https://www.cuh.nhs.uk/patient-information/information-for-patients-who-are-having-a-ct-scan/ (accessed on 1 November 2023)
Optician—eye test	£25	Specsavers prices [49]	Per eye test. Private eye tests were assumed to last 30 min [50]	https://www.specsavers.co.uk/help-and-faqs/how-much-is-an-eye-test (accessed on 1 November 2023) The College of Optometrists. https://lookafteryoureyes.org/eye-examinations/the-eye-examination/#:~:text=The%20eye%20examination%20usually%20takes,your%20general%20health (accessed on 1 November 2023)

**Medications**	**Name Given in BNF**	**Active Ingredient**	**Dose**	**Size**	**Unit**	**Cost per Item**	**Cost per Unit**	**Source**	**Notes**
Levodepa	Co-careldopa 25 mg/100 mg tablets	Co-careldopa (Carbidopa/levodopa)	3 tablet per day	1	Tablet	£20.42	£0.19	PCA 2020-21 [51]	BNF presentation code: 0409010N0AAABAB. Drugs used in parkinsonism and related disorders. At first, 25 milligrams (mg) of carbidopa and 100 mg levodopa (1 tablet) 3 times a day.
Donepezil	Donepezil 5 mg tablets	Donepezil hydrochloride	1 tablet per day	1	Tablet	£0.99	£0.04	PCA 2020-21 [51]	BNF presentation code: 0411000D0AAAAAA. Dementia medication, 5 mg per day

**Other (Patient Costs)**	**Unit**	**Cost**	**Details**	**Cost Year**	**Source**	**Notes**	**Conversion**	**Inflation**
Mileage	Mile	£0.45	NA	2021	HMRC Mileage rates [52]	Travel—mileage and fuel rates and allowances https://www.gov.uk/government/publications/rates-and-allowances-travel-mileage-and-fuel-allowances/travel-mileage-and-fuel-rates-and-allowances (accessed on 8 October 2023)	NA	NA
Opportunity costs—loss of earnings (base case analysis)	Hourly rate	£8.91	National Living Wage for workers ages 23 and over	2021	[34]	Welsh Government (2021) https://www.gov.uk/national-minimum-wage-rates (accessed on 22 June 2023)	NA	NA
Opportunity costs—lost leisure time (sensitivity analysis)	Per hour	16 Euros	An hour of leisure time lost valued at 16 Euro in cost year 2014	2014	[35]	Verbooy, K., Hoefman, R., Van Exel, J. and Brouwer, W., 2018. Time is money: investigating the value of leisure time and unpaid work. Value in Health, 21(12), pp.1428–1436.	Converted to £13.26 in 2014 using the International Monetary Fund.	Inflated to £14.82 using Curtis NHSCII Pay & Prices
			(na) Not applicable.					

## Appendix C

**Table A1:** Initial contact costs (lost leisure time forgone valued at £14.82 per hour [35].

Participant	Initial Contact (NHS/Private)	Health Service Activity	Unit Cost:	Consultation Time (Minutes)	Travel Time (Minutes)	Opportunity Costs—Patient Consultation Time (£)	Opportunity Costs—Patient Travel Time (£)	Patient Funded Costs—Mileage (£0.45 per Mile)	Total Costs (£)
Participant 1	NHS	GP consultation	£39.23	9.22	**	£2.28	**	**	£41.51
Participant 2	NHS	Neurologist consultation	£120	60	**	£14.82	**	**	£134.82
Participant 3	NHS	Ophthalmology appointment	£165.95	480 ***	30	£118.56	£7.41	**	£291.92
Participant 4	Private	Consultant neurologist appointment	£250	30	**	£7.41	**	**	£257.41
Participant 5	NHS	GP consultation	£39.23	9.22	**	£2.28	**	**	£41.51
Participant 6	NHS	GP consultation	£39.23	9.22	**	£2.28	**	£1.35	£42.86
Participant 7	NHS	GP consultation	£39.23	9.22	**	£2.28	**	**	£41.51
Participant 8	NHS	GP consultation	£39.23	9.22	**	£2.28	**	**	£41.51
Participant 9	NHS	GP consultation	£39.23	9.22	**	£2.28	**	**	£41.51
Participant 10	NHS	GP consultation	£39.23	9.22	**	£2.28	**	**	£41.51
Total cost for NHS consultations:			£561						
Total cost for private consultations:			£250						
**Totals:**			**£811**	**634.54**	**30**	**£156.75**	**£7.41**	**£1.35**	**£976**

(**) Missing data. (***) Consultation time information provided by the participant.

**Table A2:** Initial referral costs (lost leisure time forgone valued at £14.82 per hour [35].

Participant	Initial Referral (NHS/Private)	Health Service Activity	Unit Cost	Consultation Time (Minutes)	Travel Time (Minutes)	Opportunity Costs—Patient Consultation Time (£)	Opportunity Costs—Patient Travel Time (£)	Patient Funded Costs—Mileage (£0.45 per Mile)	Total Costs (£)
Participant 1	NHS	CT scan	£70.00	60	20	£14.82	£4.94	**	£90
Participant 2	NHS	MRI scan	£110.00	60	**	£14.82	**	**	£125
Participant 3	NHS	Ophthalmology follow-up appointment	£139.23	240 ***	30	£59.28	£7.41	**	£206
Participant 4	NHS	Community mental health nurse contact	£44.00	60	**	£14.82	**	**	£59
Participant 5	NHS	CT scan	£70.00	60	**	£14.82	**	**	£85
Participant 6	NHS	Community mental health nurse contact	£44.00	60	**	£14.82	**	£1.35	£60
Participant 7	NHS	Well woman appointment	£44.00	60	**	£14.82	**	**	£59
Participant 8	NHS	Memory clinic appointment	£528.00	60	**	£14.82	**	£6.30	£549
Participant 9	NHS	Psychiatrist consultation	£120.00	60	**	£14.82	**	£1.80	£137
Participant 10	NHS	CT scan	£70.00	60	**	£14.82	**	**	£85
**Totals:**			**£1239**	**780**	**50**	**£193**	**£12.35**	**£9.45**	**£1454**

(**) Missing data. (***) Consultation time information provided by the participant.

**Table A3:** Further NHS referral and activity costs (lost leisure time forgone valued at £14.82 per hour [35].

Participant	Health Service Activity	Unit Cost	Consultation Time (Minutes)	Travel Time (Minutes)	Opportunity Costs—Patient Consultation Time (£)	Opportunity Costs—Patient Travel Time (£)	Patient Funded Costs—Mileage (£0.45 per Mile)	Total Costs (£)
Participant 1	GP telephone call	£15.52	**	na	**	na	na	£16
	Memory service telephone call	£8.67	**	na	**	na	na	£9
	Consultation neurologist consultation	£123.00	60	**	£14.82	**	**	£138
Participant 2	Psychologist consultation	£65.00	60	**	£14.82	**	**	£80
	Memory service appointment (June 2015)	£528.00	60	40	£14.82	£9.88	**	£553
	Memory service appointments x12 (December 2015–June 2021)	£6336.00	720	480	£177.84	£118.56	**	£6632
	SPECT scan	£243.16	60	120	£14.82	£29.64	**	£288
	MRI scan	£110.00	60	120	£14.82	£29.64	**	£154
	Consultation with consultant psychologist and consultant psychiatrist	£243.00	60	40	£14.82	£9.88	**	£268
Participant 3	MRI scan	£110.00	60	**	£14.82	**	**	£125
	Ophthalmology appointment (follow-up)	£139.23	60	30	£14.82	£7.41	**	£161
	Ophthalmology appointment (follow-up)	£139.23	60	30	£14.82	£7.41	**	£161
	GP consultation	£39.23	9.22	**	£2.28	**	**	£42
	Neurologist consultation	£120.00	60	30	£14.82	£7.41	**	£142
Participant 4	Levadopa. Parkinsons medication (2-week trial)	£7.98	na	na	na	na	na	£8
	Neurologist consultation	£120.00	60	120	£14.82	£29.64	**	£164
Participant 5	-	-	-	-	-	-	-	-
Participant 6	GP consultation	£39.23	9.22	**	£2.28	**	£1.35	£43
	Psychologist consultation	£65.00	60	**	£14.82	**	£6.30	£86
	MRI scan	£110.00	60	**	£14.82	**	£7.20	£132
	Psychiatrist consultation	£120.00	60	**	£14.82	**	£7.20	£142
	Psychiatrist phone call	£8.67	**	na	**	na	na	£9
Participant 7	Psychiatrist consultation	£120.00	60	**	£14.82	**	**	£135
	MRI scan	£110.00	60	**	£14.82	**	£39.60	£164
	Psychiatrist consultation	£120.00	60	10	£14.82	£2.47	**	£137
	Mental health nurse	£41.00	60	**	£14.82	**	**	£56
	Donepezil 5 mg (1 day)	£0.04	na	na	na	na	na	£0
	Psychiatrist phone call	£8.67	**	na	**	na	na	£9
Participant 8	CT scan	£70.00	60	**	£14.82	**	**	£85
	Consultant psychiatrist consultation	£123.00	60	**	£14.82	**	£8.10	£146
Participant 9	Psychologist consultation	£65.00	60	**	£14.82	**	**	£80
	Psychologist consultation	£65.00	60	**	£14.82	**	**	£80
	Psychiatrist consultation (x8)	£960.00	480	**	£118.56	**	£14.40	£1093
	Psychologist home visit (2 h)	£130.00	120	**	£29.64	**	**	£160
	MRI scan	£110.00	60	**	£14.82	**	**	£125
	Psychiatrist consultation	£120.00	60	**	£14.82	**	£1.80	£137
Participant 10	Neurologist consultation	£120.00	60	**	£14.82	**	**	£135
**Totals:**		**£10,854**	**2838**	**1020**	**£701**	**£252**	**£86**	**£11,893**

(**) Missing data. (na) Not applicable. (-) No consultations were reported.

**Table A4:** Further private consultation and activity costs (lost leisure time forgone valued at £14.82 per hour [35].

Participant	Health Service Activity	Unit Cost	Consultation Time (Minutes)	Travel Time (Minutes)	Opportunity Costs—Patient Consultation Time (£)	Opportunity Costs—Patient Travel Time (£)	Patient Funded Costs—Mileage (£0.45 per Mile)	Total Costs (£)
Participant 1	Neurologist consultation	£250	30	120	£7.41	£29.64	**	£287
	MRI scan	£395	60	120	£14.82	£29.64	**	£439
	Neurologist online consultation (Zoom)	£250	30	na	£7.41	na	na	£257
Participant 2	-	-	-	-	-	-	-	-
Participant 3	Optician eye test ×2	£50	60	**	£14.82	**	**	£65
	Optician eye test ×2	£50	60	**	£14.82	**	**	£65
Participant 4	-	-	-	-	-	-	-	-
Participant 5	Neurologist consultation	£250	30	**	£7.41	**	£10.80	£268
	Neurologist consultation	£250	30	**	£7.41	**	£10.80	£268
Participant 6	CT scan	£695	60	**	£14.82	**	£7.20	£717
Participant 7	-	-	-	-	-	-	-	-
Participant 8	-	-	-	-	-	-	-	-
Participant 9	-	-	-	-	-	-	-	-
Participant 10	Neurologist consultation	£250	30	**	£7.41	**	**	£257
**Totals:**		**£2440**	**390**	**240**	**£96**	**£59**	**£29**	**£2624**

(**) Missing data. (na) Not applicable. (-) No consultations were reported.

**Table A5:** Total costs of diagnosis pathway (lost leisure time forgone valued at £14.82 per hour [35].

Patient	NHS Costs	Patient Funded Costs—Private Consultations	Travel Time (Minutes)	Consultation Time (Minutes)	Opportunity Costs—Travel Time	Opportunity Costs—Patient Consultation Time	Patient Funded Costs—Mileage	Total
Participant 1	£256.42	£895	260	249.22	£64.22	£61.56	**	£1277
Participant 2	£7755.16	£0	800	1140	£197.60	£281.58	**	£8234
Participant 3	£852.87	£100	150	1089.22	£37.05	£269.04	**	£1259
Participant 4	£171.98	£250	120	150	£29.64	£37.05	**	£489
Participant 5	£109.23	£500.00	**	129.22	**	£31.92	£21.60	£663
Participant 6	£426.13	£695.00	**	318.44	**	£78.66	£31.95	£1232
Participant 7	£482.94	£0.00	10	309.22	£2.47	£76.38	£39.60	£601
Participant 8	£760.23	£0.00	**	189.22	**	£46.74	£14.40	£821
Participant 9	£1609.23	£0.00	**	909.22	**	£224.58	£18.00	£1852
Participant 10	£229.23	£250.00	**	159.22	£0.00	£39.33	£0.00	£519
**Total**	**£12,653**	**£2690**	**1340**	**4643**	**£331**	**£1147**	**£126**	**£16,947**

(**) Missing data.

## References

[1] Gauthier S., Rosa-Neto P., Morais J.A., Webster C. (2021). World Alzheimer Report 2021: Journey though the Diagnosis of Dementia.

[2] Alzheimer’s Society. https://www.alzheimers.org.uk/.

[3] Alzheimer’s Research UK (2020) Dementia Statistics Hub: Diagnoses in the UK. https://www.dementiastatistics.org/statistics/diagnoses-in-the-uk/.

[4] Harvey R.J., Roques P., Fox N.C., Rossor M.N. (2003). Non-Alzheimer dementias in young patients. Br. J. Psychiatry.

[5] Murray M.E., Graff-Radford N.R., Ross O.A., Petersen R.C., Duara R., Dickson D.W. (2011). Neuropathologically defined subtypes of Alzheimer’s disease with distinct clinical characteristics: A retrospective study. Lancet Neurol..

[6] Woolley J.D., Khan B.K., Murthy N.K., Miller B.L., Rankin K.P. (2011). The diagnostic challenge of psychiatric symptoms in neurodegenerative disease; rates of and risk factors for prior psychiatric diagnosis in patients with early neurodegenerative disease. J. Clin. Psychiatry.

[7] Brotherhood E.V., Stott J., Windle G., Barker S., Culley S., Harding E., Camic P.M., Caufield M., Ezeofor V., Hoare Z. (2020). Protocol for the Rare Dementia Support Impact study: RDS Impact. Int. J. Geriatr. Psychiatry.

[8] Rare Dementia Support. https://www.raredementiasupport.org/.

[9] Hendriks S., Peetoom K., Bakker C., van der Flier W.M., Papma J.M., Koopmans R., Verhey F.R.J., de Vugt M., Köhler S., Withall A. (2021). Global Prevalence of Young-Onset Dementia: A systematic review and meta-analysis. JAMA Neurol..

[10] Allen J., Oyebode J.R., Allen J. (2009). Having a father with young onset dementia: The impact on well-being of young people. Dementia.

[11] Gelman C., Rhames K. (2020). “I have to be both mother and father”: The impact of Young-onset dementia on the partner’s parenting and the children’s experience. Dementia.

[12] Svanberg E., Spector A., Stott J. (2011). The impact of young onset dementia on the family: A literature review. Int. Psychogeriatr..

[13] van Vliet D., de Vugt M.E., Bakker C., Koopmans R.T.C.M., Verhey F.R.J. (2010). Impact of early onset dementia on caregivers: A review. Int. J. Geriatr. Psychiatry.

[14] Sullivan M.P., Williams V., Grillo A., McKee-Jackson R., Camic P.M., Windle G., Stott J., Brotherhood E., Crutch S.J., on behalf of the Rare Dementia Support (RDS) Research Team (2020). Peer support for people living with rare or young onset dementia: An integrative review. Dementia.

[15] Department of Health Prime Minister’s Challenge on Dementia 2020 Implementation Plan. https://assets.publishing.service.gov.uk/government/uploads/system/uploads/attachment_data/file/507981/PM_Dementia-main_acc.pdf.

[16] Welsh Government Dementia Action Plan for Wales 2018–2022. https://gov.wales/sites/default/files/publications/2019-04/dementia-action-plan-for-wales.pdf.

[17] National Institute for Health and Care Excellence Dementia—Assessment, Management and Support for People Living with Dementia and Their Carers. https://www.nice.org.uk/guidance/indevelopment/gid-cgwave0792/consultation/html-content-3.

[18] Parker M., Barlow S., Hoe J., Aitken L. (2020). Persistent barriers and facilitators to seeking help for a dementia diagnosis: A systematic review of 30 years of the perspectives of carers and people with dementia. Int. Psychogeriatr..

[19] Chithiramohan A., Iliffe S., Khattak I. (2019). Identifying barriers to diagnosing dementia following incentivisation and policy pressures: General practitioners’ perspectives. Dementia.

[20] Judge D., Roberts J., Khandker R., Ambegaonkar B., Black C.M. (2019). Physician Perceptions about the Barriers to Prompt Diagnosis of Mild Cognitive Impairment and Alzheimer’s Disease. Int. J. Alzheimer’s Dis..

[21] Bailey C., Dooley J., McCabe R. (2018). ‘How do they want to know?’ Doctors’ perspectives on making and communicating a diagnosis of dementia. Dementia.

[22] Merl H., Doherty K.V., Alty J., Salmon K. (2022). Truth, hope and the disclosure of a dementia diagnosis: A scoping review of the ethical considerations from the perspective of the person, carer and clinician. Dementia.

[23] Poyser C.A., Tickle A. (2018). Exploring the experience of the disclosure of a dementia diagnosis from a clinician, patient and carer perspective: A systematic review and Meta-ethnographic synthesis. Aging Ment. Health.

[24] Landeiro F., Wace H., Ghinai I., Nye E., Mughal S., Walsh K., Roberts N., Lecomte P., Wittenberg R., Wolstenholme J. (2018). Resource utilisation and costs in predementia and dementia: A systematic review protocol. BMJ Open.

[25] Michalowsky B., Flessa S., Hertel J., Goetz O., Hoffmann W., Teipel S., Kilimann I. (2017). Cost of diagnosing dementia in a German memory clinic. Alzheimer’s Res. Ther..

[26] Prince M., Knapp M., Guerchet M., McCrone P., Prina M., Comas-Herrera M., Wittenberg A., Adelaja R., Hu B., King B. Dementia UK: Update. Alzheimer’s Society 2014. http://www.alzheimers.org.uk/dementiauk-.

[27] Henley J., Hillman A., Jones I.R., Woods B., MacLeod C.A., Pentecost C., Clare L. (2021). ‘We’re happy as we are’: The experience of living with possible undiagnosed dementia. Ageing Soc..

[28] Kallio H., Pietilä A., Johnson M., Kangasniemi M. (2016). Systematic methodological review: Developing a framework for a qualitative semi-structured interview guide. J. Adv. Nurs..

[29] Braun V., Clarke V. (2006). Using thematic analysis in psychology. Qual. Res. Psychol..

[30] Braun V., Clarke V. (2014). What Can “Thematic Analysis” Offer Health and Wellbeing Researchers?. Int. J. Qual. Stud. Health Wellbeing.

[31] Charles J.M., Edwards R.T., Bywater T., Hutchings J. (2013). Micro-Costing in Public Health Economics: Steps Towards a Standardized Framework, Using the Incredible Years Toddler Parenting Program as a Worked Example. Prev. Sci..

[32] Edwards R.T., McIntosh E. (2019). Applied Health Economics for Public Health Practice and Research.

[33] Guy M. (2019). Between ‘going private’ and ‘NHS privatisation’: Patient choice, competition reforms and the relationship between the NHS and private healthcare in England. Leg. Stud..

[34] UK Government National Minimum Wage and National Living Wage rates. UK Government. https://www.gov.uk/national-minimum-wage-rates.

[35] Verbooy K., Hoefman R., van Exel J., Brouwer W. (2018). Time Is Money: Investigating the Value of Leisure Time and Unpaid Work. Value Health.

[36] (2005). Mental Capacity Act. Department of Health. London, HMSO. https://www.legislation.gov.ukukpga/2005/9/contents/enacted.

[37] Bayly M., O’connell M.E., Kortzman A., Peacock S., Morgan D.G., Kirk A. (2021). Family carers’ narratives of the financial consequences of young onset dementia. Dementia.

[38] Frost R., Walters K., Wilcock J., Robinson L., Dening K.H., Knapp M., Allan L., Rait G. (2021). Mapping post-diagnostic dementia care in England: An e-survey. J. Integr. Care.

[39] Giebel C., Hanna K., Tetlow H., Ward K., Shenton J., Cannon J., Butchard S., Komuravelli A., Gaughan A., Eley R. (2021). “A piece of paper is not the same as having someone to talk to”: Accessing post-diagnostic dementia care before and since COVID-19 and associated inequalities. Int. J. Equity Health.

[40] Stamou V., La Fontaine J., O’malley M., Jones B., Gage H., Parkes J., Carter J., Oyebode J. (2020). The nature of positive post-diagnostic support as experienced by people with young onset dementia. Aging Ment. Health.

[41] Rabionet S. (2011). How I Learned to Design and Conduct Semi-structured Interviews: An Ongoing and Continuous Journey. Qual. Rep..

[42] Stuckey H.L. (2013). Three types of interviews: Qualitative research methods in social health. J. Soc. Health Diabetes.

[43] Cachia M., Millward L. (2011). The telephone medium and semi-structured interviews: A complementary fit. Qual. Res. Organ. Manag. Int. J..

[44] Jones K.C., Burns A. Unit costs of health and social care 2021 Personal Social Services Research Unit. https://www.pssru.ac.uk/project-pages/unit-costs/unit-costs-of-health-and-social-care-2021/.

[45] National Schedule of NHS costs 2020–21. https://www.england.nhs.uk/publication/2020-21-national-cost-collection-data-publication/.

[46] NHS National Tariff Workbook 2020/21 (Annex A). https://www.england.nhs.uk/wp-content/uploads/2021/02/20-21NT_Annex_A_National_tariff_workbook.xlsx.

[47] Information for patients who are having a CT scan. https://www.cuh.nhs.uk/patient-information/information-for-patients-who-are-having-a-ct-scan/.

[48] Nuffield Health Hospital Guide Price. https://www.nuffieldhealth.com/consultants?size=n_20_n&sort-field=sortname&sort-direction=asc.

[49] Specsavers. https://www.specsavers.co.uk/help-and-faqs/how-much-is-an-eye-test.

[50] The College of Optometrists. https://lookafteryoureyes.org/eye-examinations/the-eye-examination/#:~:text=The%20eye%20examination%20usually%20takes,your%20general%20health.

[51] Prescription Cost Analysis. https://www.nhsbsa.nhs.uk/statistical-collections/prescription-cost-analysis-england/prescription-cost-analysis-england-2020-21.

[52] Travel—Mileage and Fuel Rates and Allowances. HMRC. https://www.gov.uk/government/publications/rates-and-allowances-travel-mileage-and-fuel-allowances/travel-mileage-and-fuel-rates-and-allowances.

